# Loncastuximab Tesirine in the Treatment of Relapsed or Refractory Diffuse Large B-Cell Lymphoma

**DOI:** 10.3390/ijms25147580

**Published:** 2024-07-10

**Authors:** Luis Miguel Juárez-Salcedo, Santosh Nimkar, Ana María Corazón, Samir Dalia

**Affiliations:** 1 Hematology Department, La Princesa University Hospital, 28006 Madrid, Spain; luismiguel.juarez@salud.madrid.org; 2 College of Osteopathic Medicine, Kansas City University, Kansas City, MO 64804, USA; santosh.nimkar@kansascity.edu; 3 Primary Care Medicine, Adelfas Primary Care Center, 28007 Madrid, Spain; anamaria.corazon@salud.madrid.org; 4 Hematology/Oncology Department, Mercy Clinic Oncology and Hematology, Joplin, MI 73120, USA

**Keywords:** immunotherapy, antibody–drug conjugate, diffuse large-B-cell lymphoma, refractory/relapse, loncastuximab tesirine

## Abstract

Currently, a significant percentage of patients with DLBCL are refractory or relapse after a first line of immunochemotherapy. Second relapses after autologous stem cell transplantation or chimeric antigen receptor T-cell therapies present few treatment options and do not yield good results. New molecules have entered the immunotherapy arsenal. Loncastuximab tesirine comprises a humanized anti-CD19 monoclonal conjugated antibody, which consists of an anti-CD19 antibody and cytotoxic alkylating agent, SG3199. Several studies have proven its efficacy in the treatment of refractory cases of DLBCL with a good safety profile, with the main adverse effects being neutropenia, thrombopenia, and liver enzyme involvement. In this review, we explain the mechanism of action of this molecule, the clinical data that have led to its acceptance by the FDA, and the new therapeutic options that are proposed in association with this drug.

## 1. Background

DLBCL is the seventh most common cancer in the general population and the most common hematological malignancy, occurring in approximately 30% of all cases of non-Hodgkin’s lymphoma [1]. Currently, immunochemotherapy treatment with the addition of the monoclonal antibody rituximab to the combination of cyclophosphamide, doxorubicin, vincristine, and prednisone (CHOP) achieves high cure rates of about 60–65% of cases [2]. However, 10–15% of patients treated with R-CHOP are primary refractory (incomplete response or relapse in the first 6 months after treatment) [3,4]. This population achieves overall response rates (ORR) of 26% and complete response rates (CR) of 7%, with a median survival of 6.3 months [5].

Despite advances in the initial treatment of this type of high-grade lymphoma, therapeutic options in relapsed/refractory patients are limited. In fit transplant-eligible patients, treatment includes high-dose chemotherapy followed by autologous hematopoietic cell transplantation. However, only half of these patients respond to initial salvage therapy, and the overall cure rate is 35–45% after autologous transplantation [6,7].

The management of non-transplant candidates who fail to respond to salvage therapy and those who relapse after autologous bone marrow transplantation is challenging. For a long time, the only viable options included single-agent or low-dose multi-agent chemotherapy with palliative intent. Today, new therapeutic options have emerged that include immunotherapy and cell therapy.

DLBCL is a heterogeneous disease, with different clinical, morphologic, and immunohistochemical features depending on the subtype. High-grade B-cell lymphoma with MYC and BCL2 and/or BCL6 rearrangements (named double hit or triple hit) is clinically aggressive, with poor prognosis and low survival rates. Similarly, double- or triple-expressing lymphomas, as well as high-grade lymphoma transformed from indolent lymphoma or presence of TP53 mutation/deletion, are associated with poor prognosis [8]. In these subgroups, standard therapy has inferior efficacy and there is an urgent need to identify effective and well-tolerated therapeutic alternatives.

In recent years, new molecules and therapies have appeared on the scene in the treatment of relapsed or refractory DLBCL. These alternatives seek to bring some hope to the dark outlook for survival in this type of patient.

The most important advance in recent years has been CD19-targeted chimeric antigen receptor-modified T-cell (CART) therapy [9]. In 2017, this therapy gained Food and Drug Administration (FDA) approval for treatment of relapsed refractory DLBCL due to durable response rates in approximately 40% of recipient patients. However, this therapy is not without serious side effects, such as cytokine release syndrome or neurological toxicity [10].

Novel monoclonal antibodies, bispecific antibodies against T cells (BiTE), and antibody–drug conjugates (ADCs) are new therapeutic options that have appeared on the treatment horizon for these relapsed/refractory patients and are the focus of research attention. ADCs are immunoconjugated molecules consisting of a modified monoclonal antibody chemically linked to a cytotoxic drug by a stable chemical linker. The final molecule is able to precisely deliver a cytotoxic drug to the desired target cell, resulting in a greater effect on the tumor with fewer side effects [11].

After antigen binding, the antibody–drug conjugate (ADC) is internalized through the process of receptor-mediated endocytosis, followed by degradation of the linker and release of the cytotoxic molecule. Finally, direct DNA damage results in cell death through direct damage by the payload or through disruption of cellular processes such as tubulin polymerization and polypeptide synthesis.

Since 2009, the FDA has approved several treatments, including the CD79a-targeted ADC, polatuzumab vedotin-piiq, in combination with bendamustine and rituximab (Pola-BR) [12]. Other regimens including drugs such as Selinexor [13], an XPO1 inhibitor, have been approved since June 2020; tafasitamab-cxix, a humanized anti-CD19 monoclonal antibody, in combination with lenalidomide, received approval in July 2020 [14]. More recently, the ADC loncastuximab tesirine-lpyl was approved in April 2021 [15]. Several other drugs are in the pipeline for approval and others have already been approved for the management of this type of refractory lymphoma, including bispecific antibodies directed against CD20 (mosunetuzumab, glofitamab, odronextamab, epcoritamab) and ADCs such as denintuzumab mafadot targeting CD74 and inotuzumab ozogamicin targeting CD22 [16,17].

Loncastuximab tesirine is a two-component ADC, a humanized anti-CD19 monoclonal antibody conjugated to a pyrrolobenzodiazepinedimer toxin. This humanized IgG1 monoclonal antibody targets CD19, which is expressed on B-lymphocytes from pre-B to mature B-lymphocytes and is not normally expressed on other human cell lines [18,19,20]. This molecule was approved by the FDA in April 2021 for the treatment of R/R large B-cell lymphoma (including DLBCL arising by low-grade lymphoma) in adult patients after two or more lines of immune chemotherapy, based on LOTIS-2 data in 2020 [15].

Currently, the efficacy and safety of the use of this ADC in patients with R/R DLBCL has already been published by different working groups, either in monotherapy or in association with other drugs such as Ibrutinib. However, new combinations are being investigated with the aim of improving treatment options for this group of patients with few treatment alternatives.

## 2. Mechanism of Action

**Figure 1:**
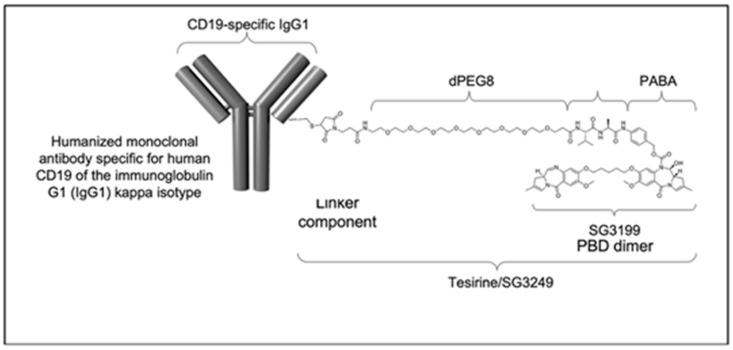
Structure of Loncastuximab.

Loncastuximab tesirine (ADCT-402, also known as Zynlonta™) is an antibody–drug conjugate (ADC) consisting of a targeted monoclonal antibody, a linker, and a cytotoxic drug [21,22,23,24]. Its humanized IgG1 monoclonal antibody targets CD19, which is expressed on B cells from pre-B to mature B cells. The antibody is conjugated to a novel cytotoxic “warhead”, the alkylating agent pyrrolobenzodiazepine dimer (PBD), via a linker that enables cathepsin removal. The combination of the cytotoxic PBD alkylating agent and SG3199 is called tesirine [21]. Unlike traditional chemotherapy, an ADC such as loncastuximab tesirine has highly selective activity, limiting toxicity and improving tolerability (Figure 1).

**Figure 2:**
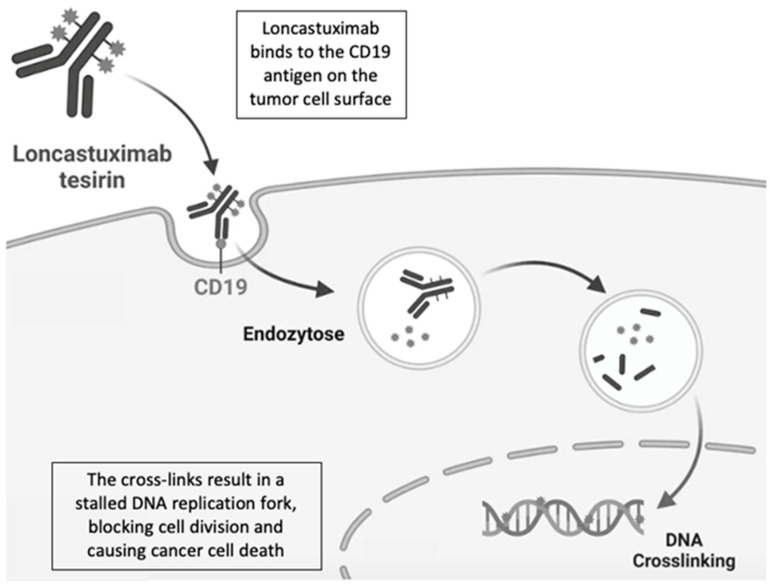
Mechanisms of action of Loncastuximab.

After binding to human CD19, loncastuximab tesirine is internalized into the cell and delivers the PBD dimer into the cell [25]. These monomers bind in the DNA minor groove and form a single covalent aminal linkage to the exocyclic N2 amino group of guanine within purine–guanine–purine sequences. PBD dimers, formed by joining two PBD monomers together via an appropriate polymethylene tether, can produce two covalent bonds forming highly cytotoxic DNA inter-strand crosslinks, preventing the separation of opposing strands, thereby disrupting basic DNA processes such as replication and ultimately inducing cell death [20] (Figure 2).

In in vitro studies, a weak but clear negative correlation (r = −0.7, *p* = 0.024) was observed between cell surface CD19 density and IC50 values in each cell line. In contrast, no relationship was observed between cell surface CD19 density and the IC50 value of SG3199 (r = −0.44, *p* = 0.2), although it showed potent cytotoxicity against the cell lines tested, consistent with its non-targeted properties. In B-cell lymphoma, the in vitro activity of loncastuximab is related to the protein expression of its target on the cell surface (*p* < 0.05) and to the RNA level of its target (*p* < 0.001). Loncastuximab had a synergistic effect with idelalisib, venetoclax and bendamustine, providing a rationale for further clinical development of different combinations.

In terms of pharmacokinetics, after a single dose of 1.5 mg/kg loncastuximab tesirine, the half-lives of the PBD conjugated antibody and the total antibody were 10.4 days and 9.9 days, respectively, confirming that this molecule has excellent in vivo stability. The results of the different clinical studies (LOTIS-1, LOTIS-2, and LOTIS-3) showed that the pharmacokinetic exposure of the loncastuximab tesirine pyrrolobenzodiazepine-conjugated antibody and the total antibody were similar, and both showed good serum stability after the first dose and after dose reduction from 150 μg/kg to 75 μg/kg at the end of cycle 2, showing sustained exposure and moderate accumulation in the 3-week regimen [21,26].

## 3. Clinical Trial Data

The potential efficacy of loncastuximab tesirine (loncastuximab/ADCT-402/MT-2111) in the treatment of Diffuse Large B-Cell Lymphoma (DLBCL) was initially elucidated in the LOTIS-1 trial (NCT02669017) for relapsing/remitting B-cell Non-Hodgkin Lymphoma (r/r B-NHL). A total of 41.3% of these DLBCL patients had relapsed after a prior response to a therapy line and 57.1% had been refractory to the last therapy line [24].

Researchers found an overall response rate (ORR) of 54.9% (28 of 51) with a complete response rate (CRR) of 37.3% (19 of 51), and a partial response rate (PRR) of 17.6% (9 of 51) in patients with relapsed/refractory DLBCL (r/r DLBCL) that received doses of ≥120 µg/kg [24]. Overall survival (OS) and progression free survival were determined to be 10.1 months and 2.9 months, respectively [24].

The majority of these patients also had tumor regression (38 of 54; 70.4%) and, post-follow-up, had a durable response in patients with CR and a median duration of response (DOR) of 3.1 months in PR patients [24]. Loncastuximab had varying levels of effectiveness with a 69.2% ORR in patients that had response to their previous therapy line and a 32.4% response in patients refractory to their previous therapy line. When subdivided, 26.5% of refractory patients (9 of 34) had a CR, 38.5% of the relapsed patients (10 of 26) had a CR, and 16.7% of patients with bulky disease (1 of 6) had a CR [24]. With these largely encouraging preliminary results, further clinical trials began testing the efficacy of loncastuximab in r/r DLBCL.

The LOTIS-2 trial (NCT02669017) was the first major human study officially conducted to assess the effectiveness of loncastuximab in treating r/r DLBCL. A total of the 139 of the 182 patients (76%) in the LOTIS-2 trial had DLBCL that had failed a median of three prior treatments (range: 1–13) [23]. ORR was 42.3% (95% CI: 33.9 to 51.1) and greater in higher doses of loncastuximab with lower doses of 15–90 µg/kg achieving lower ORR (29.4% ORR) than the higher doses of 120–200 µg/kg (47.2% ORR) [23]. It took a median time of 43 days (range: 31–323 days) for all patients to achieve a CR or PR [23]. For the majority of patients, the initial dose led to response when assessed 6 weeks/2 cycles after the treatment was started [15]. The median DOR was 4.5 months (95% CI: 3.9 to 9.5), with doses in the range of 120 µg/kg to 200 µg/kg being able to achieve similar DOR outcomes [23]. Median PFS was 2.8 months (95% CI: 1.9 to 3.8) in DLBCL patients, which was below the median of 3.1 months (95% CI: 2.7 to 4.2) in all patients. Median OS of patients with DLBCL was 7.5 months (95% CI: 6 to 9.8), which was lower than the median for all patients at 8.3 months (95% CI: 1.1,7.8) [23].

When LOTIS-2 phase 2 was completed, loncastuximab had a response rate of 48.3% in patients with r/r DLBCL [15]. Following this trial, loncastuximab became the first antibody–drug conjugate (ABD) with pyrrolobenzodiazepine (PBD), with approval for single-agent activity in r/r DLBCL and high-risk subgroups but not with bulky disease [15]. A matching-adjusted indirect comparison of efficacy concluded that loncastuximab had a significantly higher overall response rate than standard chemoimmunotherapy (CIT) for DLBCL [27]. Retrospective analyses conducted on the LOTIS-2 trial also demonstrated that younger patients (<70) and older patients (>70) appeared to have similar responses across nearly all evaluated measures [24,28]. Long-term follow-ups for the LOTIS-2 trial were conducted in September 2022, and maintained the durable responses found at the outset of LOTIS-2 phase 2 [29]. The ORR for DLBCL patients receiving loncastuximab remained the same, long-term, at 48.3% (70/145), as did the median OS and median PFS at 9.5 and 4.9 months, respectively [29]. Median DOR increased to 13.4 months. One patient with PR ended up having CR, which further increased the overall CRR to 24.8%. Of the 37 patients achieving CR, 31% and 44% were event-free for ≥1 and ≥2 years, respectively [29]. Though it remains to be seen if improved event free survival has any impact on OS and mortality, 11 of the patients that achieved CR had no evidence of disease for ≥2 years and required no additional treatment, which likely was associated with an increase in quality of life [29].

Treatment-emergent adverse events (TEAEs) were documented throughout these LOTIS trials but were managed through pre-treatment dexamethasone, spironolactone-like diuretics, and sun exposure limitations, and remained mild to moderate in severity [15,29]. Nearly all patients experienced at least one TEAE (98.6%) and the most common ones were elevated gamma glutamyl transferase (GGT) levels (42%), neutropenia (40%), and thrombocytopenia (33%). In LOTIS-1, patients had a 36.4% chance (32 of 88) of experiencing a severe TEAE of any grade and an 8% chance (7 of 88) of having a TEAE with fatal outcome. Despite this, most patients were able to tolerate at least two cycles of treatment [24]. Researchers determined that no deaths due to TEAEs were attributable to loncastuximab and no secondary malignancy or myelodysplastic syndromes were reported [29].

Table 1 summarizes the survival data from the clinical studies that led to the approval of the use of Loncastuximab in monotherapy, as well as the results of the combined use of this drug.

## 4. Toxicity Profile

Regarding the safety profile, the main emerging adverse events included hematological toxicity, fatigue, nausea, and rash. Febrile neutropenia was infrequent. The most frequent grade ≥ 3 adverse events included neutropenia, thrombocytopenia, and elevated γ-glutamyl transpeptidase (GGT) in the absence of other signs of hepatic toxicity. Hematological and hepatic toxicity of grade ≥ 3 can be controlled by withholding the drug until resolution and subsequently reducing the dose by 50% if prolonged toxicity (>3 weeks) occurs [24]. Other side effects observed were fluid retention, including pleural effusions, peripheral oedema, and pericardial effusion, which were effectively reduced by including premedication with oral dexamethasone and diuretics with spironolactone. Presumably, these are related to the PBD payload, although the pathogenic mechanisms are still unclear and may be associated with direct vascular toxicity. Rash was also a frequent adverse event and occurred mainly in sun-exposed areas. There were no cases of tumor lysis syndrome or tumor exacerbation.

## 5. Future Clinical Trials

Future LOTIS trials will seek to expand the understanding of loncastuximab efficacy in DLBCL. LOTIS-3 (NCT03684694) assessed the pairing of loncastuximab and Ibrutinib in DLBCL patients. The preliminary results from the phase 2, open-label, single-arm study had an ORR of 57.1% (95% CI: 39.4% to 73.7%), overall CRR of 34.3% (95% CI: 19.1% to 52.2%), and median DOR of 5.49 months. However, there were different outcomes for non-germinal center b-cell-like DLBCL (non-GCB DLBCL) and GCB DLBCL groups, with non-GCB achieving a lower CRR. While the DLBCL cohorts had an ORR of 57.1%, 34% CRR, and 22% PRR, the non-GCB group had a 45% ORR and 27% CRR, and the GCB group had a 77% ORR and 46% CRR [30]. This trial was subsequently terminated by an unknown administrative decision. Not many clinical trials have been conducted as of yet outside of the umbrella of the LOTIS trials. A trial that is ongoing in the Beijing Cancer Hospital (ChiCTR2300072058) replicated the study design of LOTIS-2 in a Chinese population and has shown that loncastuximab has single agent efficacy with minimal safety concerns in Chinese patients with r/r DLBCL. Their phase 2, open-label, single-arm study found that loncastuximab had an ORR of 51.6% (95% CI: 38.7% to 64.2%), CRR of 23.4%, median DOR of 6.37 months, median PFS of 4.96 months, and median OS of 9.33 months [31].

The future clinical trials will attempt to further understand loncastuximab ’s efficacy in DLBCL (Table 2). LOTIS-5 (NCT04384484), which began in September 2020 and will continue recruiting into 2028, is assessing the pairing of loncastuximab and rituximab (loncastuximab-R) vs. the standard rituximab, gemcitabine, and oxaliplatin (R-GemOx) in patients with r/r DLBCL. Preliminary results (around n ≤ 20 patients, as of now) from this phase 3 randomized controlled trial appear to show a durable response (ORR 80% [95% CI: 56.3% to 94.3%]; CRR 50% [95% CI: 27.2% to 72.8%]; PRR 30% [95% CI: 11.9% to 54.3%]; median DOR 8.02 months [95% CI: 3.19—not reached]; median PFS 8.31 months [95% CI: 4.53—not reached]) and no new safety concerns [32]. LOTIS-7 (NCT04970901) aims to investigate the efficacy of loncastuximab with various promising treatment agents including but not limited to glofitamab, mosunetuzumab, and polatuzumab vedotin in previously untreated DLBCL and will run from December 2021 to May 2027. The City of Hope Comprehensive Cancer trial (NCT05672251: January 2024–December 2024) is investigating the pairing of loncastuximab and mosunetuzumab in the treatment of r/r DLBCL. The M.D. Anderson Cancer Center is conducting a clinical trial (NCT05464719: Beginning September 2022–January 2026) to determine the efficacy of loncastuximab in patients that have achieved PR after CAR-T cell therapy. In the past, loncastuximab has shown favorable results in patients that had relapsed or had disease progression after CAR-T cell therapy [33]. Of the 13 patients in the LOTIS-2 trial that had previously had CAR-T cell therapy, 6 (46%) had responses to loncastuximab, with the median OS, PFS, and duration of response being 8.2, 1.4, and 8, respectively [33]. In patients with DLBCL that have relapsed from CAR-T cell therapy, chemotherapy was found to be ineffective [18,34]. This multicenter observational study concluded that when these patients received chemotherapy, none had CR, only 50% had PR, and the patients, overall, had poor survival metrics (6-month OS: 25% [95 CI: 11 to 59] [18,34]). There are also some case reports indicating that patients with DLBCL previously treated with loncastuximab achieved 50% ORR when given CAR-T cell therapy [18,35].

## 6. Conclusions

Loncastuximab tesirine is a promising new therapeutic option for the management of patients with relapsed or refractory DLBCL. It is currently being used in the third line of treatment. ADCs are a new class of drugs, which can deliver the active molecule directly to the target cells, thus minimizing toxicity. Compared to the cell therapy represented by CART therapy, molecules such as loncastuximab represent a suitable option by solving the problems related to the collection and processing of the targeted T cells. In addition, treatment failure represents a major clinical challenge. There are currently no comparative studies or randomized controlled trials that directly compare the efficacy and safety of loncastuximab tesirine with that of other treatments for patients with R/R DLBCL. However, the ongoing LOTIS-5 trial will reveal the efficacy of this molecule in combination with rituximab compared to immunochemotherapy in these patients. The possibility that loncastuximab can be administered in local hospitals represents an advantage (geographic, economic, social), compared to patients who are candidates for CART treatment and who require referral to more complex centers.

Further trials and follow-up studies will help to better assess its role, both in the first line and in early relapsing or refractory episodes.

## Figures and Tables

**Table 1:** Completed loncastuximab clinical trials.

Trial	N=	Dosing	OR (ORR)	CR; PR CRR; PRR	PFS; OS; Median DOR (Months)
LOTIS-1 (open-label, single-arm, phase 1 study) ^1^ [COMPLETED]	63	3 × 3 Dose-escalation design to determine adequate dose	≤90 µg/kg: 2 (20%) 120 µg/kg: 6 (54.5%) 150 µg/kg: 9 (60.0%) 200 µg/kg: 13 (52.0%) ≥120 µg/kg: 28 (54.9%)	≤90 µg/kg: 1; 1 120 µg/kg: 4; 2 150 µg/kg: 5; 4 200 µg/kg: 10; 3 ≥120 µg/kg: 19; 9 20 (32.8%); 10 (16.4%)	2.9 *; 10.1 DOR: CR- not reached PR-3.1 150 µg/kg: 5.5 120 µg/kg: 4.9 200 µg/kg: 4.1
LOTIS-2 (multicenter, open-label, single-arm, phase 2 study) ^2^ [COMPLETED]	145	Q3w 150 µg/kg first 2 cycles 75 µg/kg for subsequent cycles, up to 1 year	70 (48.3%) [95% CI: 39.9% to 56.7%]	35; 35 24.1% [95% CI: 17.5% to 31.9%] **; 24%	4.9 [95% CI: 2.9–8.3]; 9.9 *** [95% CI: 6.7–11.5]; 10.3 **** [95% CI: 6.9-not reached]
LOTIS-3 ^x^ (loncastuximab + Ibrutinib: Phase 2 open-label, single-arm study) ^3^ [TERMINATED]	35	60 µg/kg loncastuximab q3w for 2 cycles + 560mg/day Ibrutinib oral up to 1 year (CR/PR/stable disease patients receive additional loncastuximab cycle 5,6,9,10)	20 (57.1%) [95% CI: 39.4% to 73.7%]	12; 8 34.3% [95% CI: 19.1% to 52.2%]; 22.9% #	PFS not reported; OS not reported; 5.49 [95% CI: 6.9- not reached

* This is a primary endpoint result. Secondary endpoint result: 7.5-month PFS [27]. ** This is a primary endpoint result. Secondary endpoint result: 24.8% CRR [15]. *** This is a primary endpoint result. Secondary endpoint result: 9.5-month OS [15]. **** This is a primary endpoint result. Secondary endpoint result: 13.4-month median DOR, median not reached [15]. # No CI was provided. ^x^ This study was terminated. ^1^ Data are from Kahl et al. 2019 [24]. ^2^ Data are from Caimi et al. 2021 [15]. ^3^ Data are from Carlo-stella et al. 2021 [30]. All remaining trial information can be found on the publicly available clinical trials government website.

**Table 2:** Ongoing loncastuximab clinical trials.

Trial	Trial Identification Number and Trial Dates	N=	Any Available Results
LOTIS-5 (loncastuximab + R-GemOx [rituximab–gemcitabine–oxaliplatin]) ^1^	NCT04384484 September 2020– 2028	~330 by trial end. Currently n ≤ 20	Preliminary Data: OR(ORR): 16/20 (80%) [95% CI: 56.3% to 94.3%] CRR; PRR: 50% [95% CI: 27.2% to 72.8%]; 30% [95% CI: 11.9% to 54.3%] PFS; OS; Median DOR (months): 8.31 [95% CI: 4.53—not reached]; OS not reported; 8.02 [95% CI: 3.19—not reached]
LOTIS-7 (loncastuximab + various other agents including but not limited to polatuzumab vedotin, glofitamab, and mosunetuzumab for r/r DLBCL)	NCT04970901 December 2021 - May 2027	~n = 200 (which includes patients with r/r B-NHL, DLBLCL, HGBCL, FL, MZL)	N/A
Chinese Cancer Centers Trial (Replication of LOTIS-2) ^2^	ChiCTR2300072058 September 2021 - Ongoing (unknown completion)	~n = 64 (as of 11 January 2023 data cutoff)	ORR: 51.6% [95% CI: 38.7% to 64.2%] CRR: 23.4% [NO CI provided] PFS; OS; Median DOR: 4.96; 9.33; 6.37 [NO CI provided]
MD Anderson (loncastuximab as consolidation therapy in r/r DLBCL)	NCT05464719 September 2022 - January 2026	~n = 30 (As of February 2024)	N/A
City of Hope (loncastuximab + mosunetuzumab for r/r DLBCL)	NCT05672251 January 2024 - December 2024	~n = 36 (As of February 2024)	N/A

^1^ Data are from Kwiatek et al. 2023 [32]. ^2^ Data are from Lin et al. 2023 [31]. All remaining trial information can be found on the publicly available clinical trials government website when the trial-specific identification number is entered.

## Data Availability

Not applicable.
